# The Enhanced Inhibitory Effect of Different Antitumor Agents in Self-Microemulsifying Drug Delivery Systems on Human Cervical Cancer HeLa Cells

**DOI:** 10.3390/molecules200713226

**Published:** 2015-07-21

**Authors:** Zoltán Ujhelyi, Azin Kalantari, Miklós Vecsernyés, Eszter Róka, Ferenc Fenyvesi, Róbert Póka, Bence Kozma, Ildikó Bácskay

**Affiliations:** 1 Department of Pharmaceutical Technology, Faculty of Pharmacy, University of Debrecen, Nagyerdei körút 98, Debrecen 4032, Hungary; E-Mails: ujhelyi.zoltan@pharm.unideb.hu (Z.U.); azin.kalantari@yahoo.com (A.K.); vecsernyes.miklos@pharm.unideb.hu (M.V.); roka.eszter@pharm.unideb.hu (E.R.); fenyvesi.ferenc@pharm.unideb.hu (F.F.); 2 Department of Obstetrics and Gynecology, Faculty of Medicine, University of Debrecen, Debrecen 4032, Hungary; E-Mails: pokar@med.unideb.hu (R.P.); bence.kozma@med.unideb.hu (B.K.)

**Keywords:** HeLa, bleomycin, ifosfamide, cisplatin, SMEDDS

## Abstract

The aim of this study was to develop topical self-microemulsifying drug delivery systems (SMEDDS) containing antitumor agents (bleomycin, cisplatin and ifosfamide) and to investigate their inhibitory potential in SMEDDS on human cervical cancer HeLa cells. The physicochemical properties of cytostatic drug loaded SMEDDS were characterized. The cytotoxicity of main components of SMEDDS was also investigated. Their IC_50_ values were determined. HeLa cells were treated by different concentrations of cisplatin, bleomycin and ifosfamide alone and in various SMEDDS. The inhibitory effect on cell growth was analyzed by MTT cell viability assay. Inflammation is a driving force that accelerates cancer development. The inhibitory effect of these antitumor agents has also been tested on HeLa cells in the presence of inflammatory mediators (IL-1-β, TNF-α) as an *in vitro* model of inflamed human cervix. Significant differences in the cytotoxicity of cytostatic drugs alone and in SMEDDS have been found in a concentration-dependent manner. The self-micro emulsifying system may potentiate the effectiveness of bleomycin, cisplatin and ifosfamide topically. The effect of SMEDDS containing antitumor agents was decreased significantly in the presence of inflammatory mediators. According to our experiments, the optimal SMEDDS formulation is 1:1:2:6:2 ratios of Isopropyl myristate, Capryol 90, Kolliphor RH 40, Cremophor RH40, Transcutol HP and Labrasol. It can be concluded that SMEDDS may increase the inhibitory effect of bleomycin, ifosfamide and cisplatin on human cervical cancer HeLa cells. Inflammation on HeLa cells hinders the effectiveness of SMEDDS containing antitumor agents. Our results might ensure useful data for development of optimal antitumor formulations.

## 1. Introduction

Uterine and cervix carcinoma is the first cause of gynecological cancer mortality in developing countries [1]. Cervical cancer is well-known to be a chronic inflammatory disease of multifactorial etiology [2]. Antitumor agents have played an important role in cervical cancer therapy [3]. Cisplatin is one of the main selected antitumor agents, but the remission rate of cervical cancer in advanced stage by cisplatin alone is only 17%–21% [4]. In recent chemotherapy, cisplatin, bleomycin and ifosfamide (BIP) in combination has also been applied against inoperable cervical cancer [5]. Many active pharmaceutical ingredients (API) have been developed to improve the efficacy of chemotherapy [6,7]. Combined chemotherapy results in reduced doses of single drug, lower side effects and drug resistance [8]. Due to the serious side effects of these cytostatic drugs, it is necessary to develop modern drug delivery systems which can reduce toxicity by decreasing the dose of potent therapeutic agent [9]. Self-micro emulsifying drug delivery systems (SMEDDS) are widely used to improve the bioavailability of poorly soluble drugs by presenting and maintaining API in a dissolved state, in small droplets of oil [10]. Hence, it is capable of penetration through various biological barriers [11]. However, the topical application of these systems might also be advantageous [12]. SMEDDS are composed of a mixture of surfactant, co-surfactant and various oils. These delivery systems form oil-in-water emulsions without or upon gentle agitation. SMEDDS properties always depend on *inter alia* the surfactant properties and their mixing rates. HeLa cells are a reliable model of human cervix cancer cells [13].

In the present study, our aim is to develop topical SMEDDS incorporating different antitumor agents (bleomycin, cisplatin, and Ifosfamide) and to examine their inhibitory potential in SMEDDS in the presence of inflammatory mediators on human cervical cancer HeLa cells.

## 2. Results

### 2.1. Formulation and Evaluation of Self-Micro-Emulsifying Drug Delivery Systems

**Figure 1:**
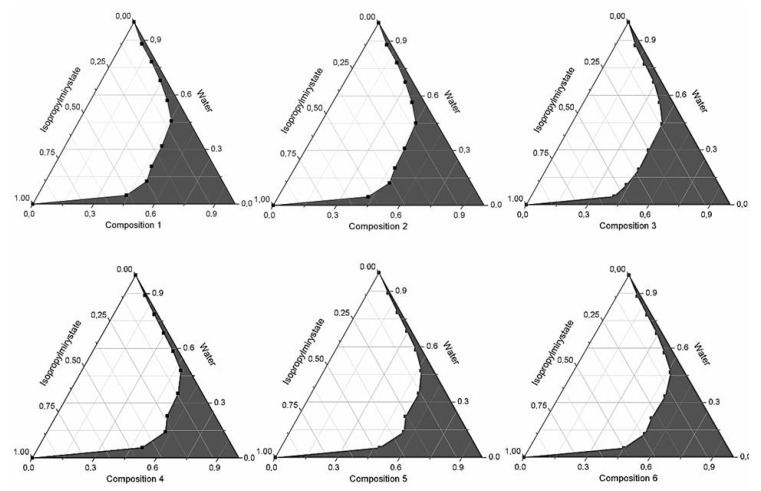
Pseudoternary phase diagrams of compositions 1–6, (Shaded areas represented microemulsions).

**Figure 2:**
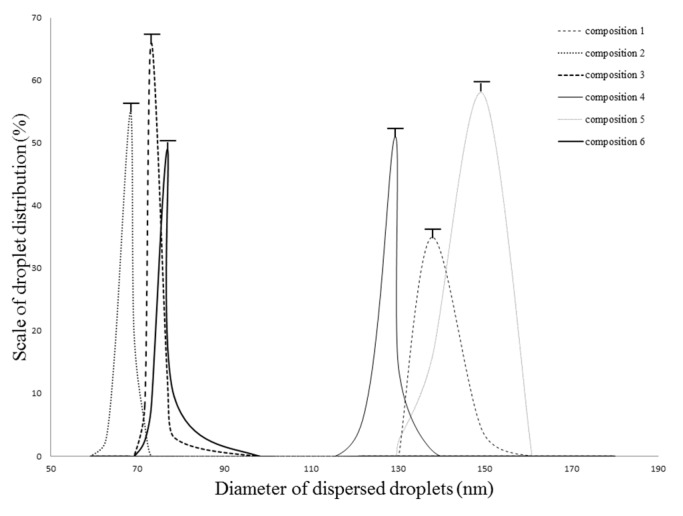
Evaluated droplet size of SMEDDS in water via DLS measurement. Evaluated average droplet sizes: composition 1: 137.81 ± 1.25 nm, composition 2: 68.44 ± 1.05 nm, composition 3: 73.28 ± 0.95 nm, composition 4: 129.31 ± 1.95 nm, composition 5: 149.35 ± 1.00 nm, composition 6: 76.90 ± 1.00 nm. Values are expressed as means ± SD, *n* = 5.

Kolliphor RH 40 and Labrasol as surfactant, Capryol 90 or Lauroglycol FCC and Transcutol HP as co-surfactants were used in our experiments. Based on the results of the pseudoternary phase diagram, every composition formed microemulsion. The maximum microemulsion existing zones observed are shown in Figure 1. The distribution of droplet size is presented in Figure 2. The developed SMEDDS (shown in Table 1) spontaneously formed microemulsion upon mild agitation in distillated water at room temperature. The percentage of transmittance and refractive index of the resulted formulation were found to be 976.8% ± 1.31% and 1.337% ± 0.13% indicating the transparency of these formulations. Moreover, the developed formulation was stable for one month at room temperature. Based on the average diameter sizes, composition 3 (1:1:2:6:2 ratio of Isopropyl myristate, Capryol 90, Kolliphor RH40 Transcutol HP and Labrasol) resulted in the smallest droplet size (78.4 nm) and the highest microemulsion zone.

**Table 1:** Ratio of surface active agents and active pharmaceutical ingredients in formulated SMEDDS.

Composition	API (mg)		Ratio of Surface Active Agents in SMEDDSs (Total Volume of Composition: 1 mL)					
			Kolliphor RH40	Labrasol	Capryol 90	Lauroglycol FCC	Transcutol HP	Isopropyl Myristate
1	0.001–0.05	Cisplatin	2	2	1	0	2	1
	0.1–5.00	Ifosfamide	2	2	1	0	2	1
	0.0005–0.05	Bleomycin sulfate	2	2	1	0	2	1
2	0.001–0.05	Cisplatin	2	2	1	0	4	1
	0.1–5.00	Ifosfamide	2	2	1	0	4	1
	0.0005–0.05	Bleomycin sulfate	2	2	1	0	4	1
3	0.001–0.05	Cisplatin	2	2	1	0	6	1
	0.1–5.00	Ifosfamide	2	2	1	0	6	1
	0.0005–0.05	Bleomycin sulfate	2	2	1	0	6	1
4	0.001–0.05	Cisplatin	1	1	0	1	1	1
	0.1–5.00	Ifosfamide	1	1	0	1	1	1
	0.0005–0.05	Bleomycin sulfate	1	1	0	1	1	1
5	0.001–0.05	Cisplatin	1	1	0	1	2	1
	0.1–5.00	Ifosfamide	1	1	0	1	2	1
	0.0005–0.05	Bleomycin sulfate	1	1	0	1	2	1
6	0.001–0.05	Cisplatin	1	1	0	1	4	1
	0.1–5.00	Ifosfamide	1	1	0	1	4	1
	0.0005–0.05	Bleomycin sulfate	1	1	0	1	4	1

Drug encapsulation efficiency was evaluated by Q. Li *et al.* method [14]. The encapsulation efficiency of our SMEDDS containing model drug was previously tested. It can be found that the encapsulation efficiency was satisfactory. This was the reason that these SMEDDS compositions were used for the formulation of these antitumor agents.

### 2.2. Droplet Size and Zeta Potential Determination

Evaluated droplet size of SMEDDS in water via DLS measurement. Evaluated average droplet sizes: composition 1: 137.81 ± 1.25 nm, composition 2: 68.44 ± 1.05 nm, composition 3:73.28 ± 0.95 nm, composition 4: 129.31 ± 1.95 nm, composition 5: 149.35 ± 1.00 nm, composition 6: 76.90 ± 1.00 nm. Every sample was found to be a monodisperse system. (Figure 2.) The zeta potential of compositions was detected to be between −5.01 ± 0.49 mV and −6.55 ± 0.099 mV. The evaluated zeta potential values were the following: composition 1: −5.91 ± 0.87 mV, composition 2: −5.82 ± 0.65 mV, composition 3: −6.55 ± 0.99, composition 4: −5.56 ± 0.32 mV, composition 5: −5.14 ± 0.71 mV, composition 6: −5.01 ± 0.49 mV. Results are reported as means ± SD, *n* = 5.

### 2.3. Cell Viability Test of SMEDDS Ingredients

**Figure 3:**
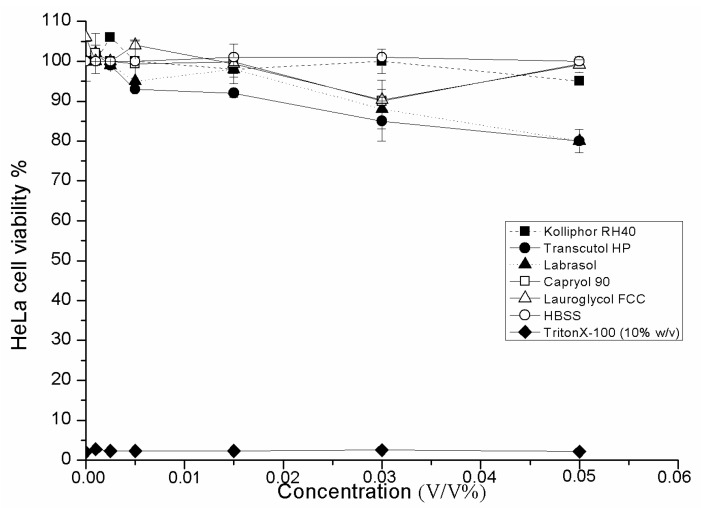
Cytotoxic effects of applied SMEDDS components (surfactant and co-surfactants) on HeLa cells determined by MTT-test. Cell viability was expressed as the percentage of untreated control in the function of surfactant concentration. Positive control: Triton X 100 (10% w/v). Values are expressed as means ± SD, *n* = 5.

Cytotoxic properties have been evaluated by MTT cell viability test. Labrasol showed the highest cytotoxicity on HeLa cells (IC_50_: 0.23 ± 0.025 v/v %), Transcutol HP was less toxic (IC_50_: 3.42 ± 0.035 v/v %), and Kolliphor RH 40 was responsible for the lowest cell viability reduction (IC_50_: 5.12 ± 0.085 v/v %). However, there are significant differences in the cytotoxic properties of surfactants in a concentration-dependent manner, the IC_50_ values of each examined surfactant is much higher than the applied concentration range in SMEDDS compositions (Figure 3).

### 2.4. Evaluation of Inhibitory Effect of SMEDDS Containing Antitumor Agent

**Figure 4:**
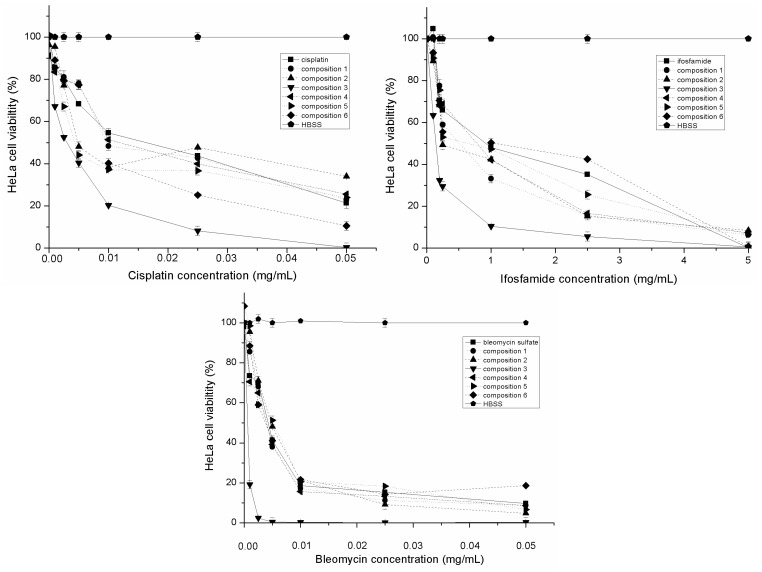
Inhibitory effect of different SMEDDS compositions containing antitumor agent (bleomycin sulfate, Ifosfamide and cisplatin) evaluated by MTT test. Cell viability was expressed as the percentage of untreated control (HBSS). Values are expressed as means ± SD, *n* = 5.

The inhibitory effects of cisplatin, bleomycin sulfate and ifosfamide alone and formulated in SMEDDS on human cervical cancer HeLa cells are depicted in Figure 4. Their calculated IC_50_ values are reported in Table 2. Based on our investigations, antitumor agents hindered the cell proliferation in a concentration-dependent manner. The SMEDDS as a carrier system can increase the antiproliferative effect of cisplatin, bleomycin sulfate and Ifosfamide. Composition 3 resulted in the highest inhibitory effect. The IC_50_ concentrations of cisplatin, blemycin and ifosfamide was mixed and incorporated in the compositions. (Table 2.) We found that the inhibitory effect of BIP combination was higher than the applied antitumor agents alone.

**Table 2:** Evaluated IC_50_ values of antitumor agents alone and in combination incorporating in different SMEDDS determined by MTT cell viability assay. Values are expressed as means ± SD, *n* = 5.

API(Bold/Compositions)	IC_50_ (μg/mL)	±SD	IC_50_ (μg/mL) in Presence of TNF-α, IL-1-β	±SD
**Cisplatin**	16.68	0.10	25.96	0.56
Composition 1	9.63	0.11	27.88	0.21
Composition 2	4.81	0.21	8.69	1.00
Composition 3	2.82	0.15	5.84	0.45
Composition 4	11.03	0.96	9.00	0.01
Composition 5	4.26	0.85	32.70	0.21
Composition 6	5.35	0.32	14.29	041
**Bleomycin sulfate**	4.21	0.11	5.69	0.11
Composition 1	3.84	0.15	4.37	0.19
Composition 2	3.99	0.50	6.70	0.32
Composition 3	0.61	0.012	0.82	0.01
Composition 4	4.71	0.12	6.81	0.01
Composition 5	5.12	0.33	7.56	0.02
Composition 6	4.93	0.22	6.33	0.01
**Ifosfamide**	993.50	25.00	1936.02	5.40
Composition 1	521.70	20.40	641.10	4.21
Composition 2	247.30	35.60	479.00	4.52
Composition 3	148.50	1.00	191.10	3.66
Composition 4	607.30	10.90	823.20	0.99
Composition 5	802.41	30.55	873.14	0.99
Composition 6	1044.70	1.11	1098.70	1.90
**BIP**	3.36	0.10	4.93	0.18
Composition 1	2.76	0.23	3.56	0.11
Composition 2	2.01	0.21	3.67	0.34
Composition 3	0.33	0.05	0.95	0.09
Composition 4	3.31	0.08	3.95	0.17
Composition 5	2.99	0.23	4.51	0.35
Composition 6	2.74	0.32	3.45	0.37

### 2.5. Evaluation of Inhibitory Effect of SMEDDS Containing Antitumor Agent in the Presence of Inflammatory Mediators

**Figure 5:**
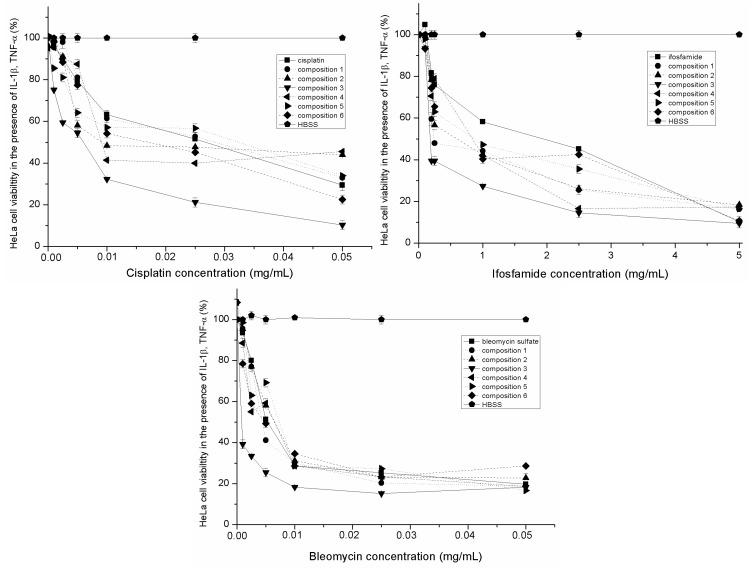
Inhibitory effect of different SMEDDS compositions containing antitumor agent (bleomycin sulfate, ifosfamide and cisplatin) in the presence of inflammatory mediators evaluated by MTT test. Cell viability was expressed as the percentage of untreated control (HBSS). Values are expressed as means ± SD, *n* = 5.

The inhibitory effect of cisplatin, bleomycin sulfate and ifosfamide alone and formulated in SMEDDS in the presence of IL-1-β and TNF-α on human cervical cancer HeLa cells are depicted in Figure 5. Their calculated IC_50_ values are also reported in Table 2. Our results revealed that the antiproliferative effect of antitumor agents in SMEDDS was decreased in the presence of inflammatory mediators. Significant differences of the IC_50_ values were determined among SMEDDS compositions. Inhibitory effect of Composition 3 resulted in the highest IC_50_ value but it was less effective in the presence of inflammatory mediators. The IC_50_ concentrations of cisplatin, blemycin and ifosfamide was mixed and incorporated in the compositions. Lower IC_50_ values were determined in the case of the mixture of antitumor agents but it was less effective in the presence of inflammatory mediators. (Table 2.).

### 2.6. Discussion 

Cervical cancer is one of the leading causes of mortality in women worldwide. Cervical screening programs have decreased the incidence of cervical cancer but the role of the most effective cytostatic therapy with less adverse effect is inevitable [15]. In our experiment, the inhibitory effect of six different SMEDDS compositions containing cisplatin, bleomycin sulfate, ifosfamide and their mixture was investigated on human cervical cancer HeLa cells. Antitumor agents: bleomycin sulfate, cisplatin and Ifosfamide in monotherapy and/or in combination seems to be adequate for the treatment of cervical cancer [16]. However, topical application of antitumor agents might be more advantageous [17]. Various topically administered SMEDDS were selected as carrier systems for the formulation of antitumor agents in our experiments. Self-microemulsifying drug delivery systems as lipid-based carriers are able to increase the solubility of poorly soluble drugs, but are also capable of modifying the permeability of different APIs by their high surfactant content as penetration enhancers [18]. The toxicity and permeability of our SMEDDS ingredients were previously tested [19]. The cytotoxicity of SMEDDS compositions was also investigated. It was confirmed that they were not toxic in the applied concentrations. The metabolic health of the cells might be maintained and inhibition of their proliferation was not a consequence of cytotoxicity of SMEDDS. Surfactants and co-surfactants could improve both the paracellular and transcellular uptake of active ingredients *in vitro* and increase their relative oral bioavailability [20]. However, many advantages of SMEDDS have been shown in oral administration, the enhanced biological effect of local therapy is still in focus [21]. Antitumor agent loaded SMEDDS systems augmented the efficacy of bleomycin sulfate, cisplatin and Ifosfamide. The highest inhibitory effect was reached by the application of composition 3 (1:1:2:6:2 ratio of Isopropyl myristate, Capryol 90, Kolliphor RH40 Transcutol HP and Labrasol). The IC_50_ concentrations of bleomycin, cisplatin and Ifosfamide were mixed and incorporated in SMEDDS. Our results showed that their inhibitory effect was additive and significantly more effective. The role of BIP in the management of advanced and recurrent carcinoma of the cervix was certified in a randomized-controlled study [22]. Orally administered bleomycin sulfate loaded nanostructured lipid particles were constructed and characterized. The *in vivo* study indicated that the safety and the potency of nanoformulation was favorable in cervical cancer cells [23]. The connection between chronical inflammation and cervical cancer was established [24]. Inflammation is a driving force that accelerates cancer development [25]. In our experiment, different SMEDDS compositions were also investigated in the presence of IL-1-β and TNF-α on human cervical cancer HeLa cells. Decreased inhibitory efficacy of these carrier systems was confirmed. This emerging evidence may indicate that inflammation can hinder the benefit of carrier systems. The regulation of inflammatory pathways was presented and the impact of external factors in the development of invasive cervical neoplastic transformation was also proved [26].

## 3. Experimental Section

### 3.1. Materials

Antitumor agents as Cisplatin (*cis*-Diammineplatinum(II) dichloride), Bleomycin sulfate, Ifosfamide (*N*,3-Bis(2-chloroethyl) tetrahydro-2*H*-1,3,2-oxazaphosphorin-2-amine-2-oxide**)** were obtained from FLUKA Analytical Ltd. ( Seelze, Germany). Labrasol, Capryol 90, Lauroglycol FCC and Transcutol HP were kind gifts from Gattefossé, Lyon, France. Kolliphor RH 40 were obtained from BASF, Ludwigshafen, Germany. 3-(4,5-dimethylthiazol-2-yl)-2,5-diphenyltetrazolium bromide (MTT), Dulbecco’s Modified Eagle’s Medium (DMEM), Hank’s Balanced Salt Solution (HBSS), phosphate buffered saline (PBS), Trypsin-EDTA, Heat-inactivated fetal bovine serum (FBS), l-glutamine, non-essential amino acids solution, penicillin-streptomycin were purchased from Sigma-Aldrich (Buchs, St Gallen, Switzerland). 96 well cell plates and culturing flasks were obtained from Corning, New York (NY, USA). IL-1-β, TNF-α have been purchased from Sigma-Aldrich. 

### 3.2. Methods

#### 3.2.1. Formulation and Evaluation of Self-Micro-Emulsifying Drug Delivery Systems

Different self-emulsifying combinations have been formulated by the water and oil dilution method with various previously tested tensides and co-tensides [27]. The compositions are listed in Table 1. Tenside components were mixed at 37 °C by Schott Tritronic dispenser (SI Analytical, Mainz, Germany) combined with Radelkis OP-912 magnetic stirrer (Radelkis, Budapest, Hungary). The applied concentrations of cytostatic drugs were dissolved in the systems at room temperature by permanent agitation. To evaluate any signs of phase separation, the mixtures were equilibrated for 24 h. An Erweka DT800 rotating paddle apparatus (Erweka Gmbh, Heusenstamm, Germany) was used to evaluate the efficiency of self-emulsification of different mixtures. One gram of each mixture was added to 200 mL of distilled water with gentle agitation condition provided by a rotating paddle at 70 rpm and at a temperature of 37 °C. The process of self-emulsification was visually monitored for the rate of emulsification and for the appearance of the produced emulsions. The visual properties registered against the increment of the applied surfactant component in Ternary triangular diagrams. Plotting points of preferential combinations were selected according to cartesian coordinate calculation.

#### 3.2.2. Droplet Size and Zeta Potential Determination

Diameter of dispersed phase was investigated by a Dynamic Light Scattering device (Malvern, Worchetershire, UK). The cumulant Dynamic Light Scattering (DLS) method was used for determination of droplet size of formulated emulsions. To obtain the diffusion coefficient the intensity correlation function has been analyzed. The measurements have been performed by Brookhaven Photometer (Brookhaven, Upton, NY, USA). During the operation temperature was 25 °C, the laser detection angle was adjusted to 90°, Lambda (λ) to 533 nm, index to 1.334 by Particle Sizing Program 3.1 (Malvern, Worchetershire, UK, 2000). Diameters of dispersed droplets according to the diffusion coefficient have been evaluated automatically by the computer program [28]. To evaluate the zeta potential the samples were diluted with 10 mL distilled water by gentle agitation at room temperature. Zeta potential of samples had been evaluated by Zetasizer NanoZS analyser (Malvern, Worchetershire, UK). Measurements were performed in quadruplets to obtain an average and standard deviation of the results. 

#### 3.2.3. Cell Culturing

HeLa (human cervical cancer cells) was obtained from the European Collection of Cell Cultures (ECACC, Public Health England, Salisbury, UK). Cells were grown in plastic cell culture flasks in Dulbecco’s Modified Eagle’s Medium (Sigma-Aldrich Buchs, St Gallen, Switzerland), supplemented with 3.7 g/L NaHCO_3_, 10% (v/v) heat-inactivated fetal bovine serum (FBS), 1% (v/v) non-essential amino acids solution, 1% (v/v) l-glutamine, 100 IU/mL penicillin, and 100 IU/mL streptomycin at 37 °C in an atmosphere of 5% CO_2_. The cells were routinely maintained by regular passaging. For cytotoxic and transport experiments, cells were used between passage numbers 20 and 40. The culture media was replaced with fresh media in every 72 h [29].

#### 3.2.4. *In Vitro* Cell Viability Assay

To exclude any toxic effect of the blank SMEDDS and their components on HeLa cells, MTT cell viability test was used [30]. Cells were seeded on flat bottom 96-well tissue culture plates at a density of 10^4^ cells/well and allowed to grow in a CO_2_ incubator at 37 °C for 4 days. For these studies, the culture medium was removed, surfactant or SMEDDS solutions were added, and the cells were incubated for a further 30 min. After removing the samples, another 3-h-incubation in a medium containing MTT at the concentration of 0.5 mg/mL followed. The dark blue formazan crystals were dissolved in acidic isopropanol (isopropanol: 1.0 N hydrochloric acid = 25:1). The absorbance was measured at 570 nm against a 690 nm reference with FLUOstar OPTIMA Microplate Reader (BMG LABTECH, Offenburg, Germany). Cell viability was expressed as the percentage of the untreated control [31].

#### 3.2.5. Inhibitory Effect of Different SMEDDS Containing Antitumor Agents

HeLa proliferation were also evaluated by using MTT cell viability assay. Cells were plated in 96-well sterile plates, at a density of 10^4^ cells per well in 100 mL of medium, and incubated for 3–4 h. Cytostatic drugs alone in cell culture medium or incorporating in SMEDDS were prepared immediately before use and added in a volume of 50 μL and total volume of 200 μL (with 150 mL fresh medium supplement) per well at final concentrations of cytostatic drugs between 5 × 10^−4^ and 5 mg/mL. After 72 h the samples were removed and 100 mL of freshly diluted MTT solution at a concentration of 0.5 mg/mL, was pipetted into each well and the plate was incubated for 3 h at 37 °C in a humidified, 5% CO_2_ atmosphere. After a specific period, cell viability was evaluated by measurement of the absorbance at 520 nm, using a FLUOstar OPTIMA Microplate Reader (BMG LABTECH). All experiments were made in quadruplicate. Standard deviations were ≤10%.

#### 3.2.6. Inhibitory Effect of Different SMEDDS Containing Antitumor Agents in the Presence of Inflammatory Mediators

The inhibitory effect of different SMEDDS containing cytostatic drugs was evaluated in the presence of inflammatory mediators on human cervical cancer HeLa cells. HeLa cells were plated in 96-well sterile plates, at a density of 10^4^ cells per well in 100 mL of medium, and incubated with 1.25 μL IL-1-β (0.1 μg/μL) and 3.75 μL TNF-α (0.1 μg/μL) for 3–4 h. After ignition, the previously described HeLa cell proliferation test was used.

#### 3.2.7. Statistical Analysis

Data were analyzed using SigmaStat (version 3.1; SPSS, IBM Inc, New York, NY, USA) and presented as means ± SD. Comparison of groups was performed by one-way ANOVA. This ANOVA was used to compare the differences of each values belong to certain concentrations in MTT. We marked the significant differences with asterisks in figures. After that, the results among the groups were presented by Tukey’s test. Differences were regarded as significant in case of *p* < 0.05. All experiments were carried out in triplicates and repeated at least three times.

## 4. Conclusions

It can be concluded that topically administered self-microemulsifying drug delivery systems containing different antitumor agents may provide an alternative treatment for cervical cancer. Inflammatory mediators can hinder the efficacy of therapy. Our results might provide useful data for development of optimal antitumor formulations.
